# Long-Lasting Novelty-Induced Neuronal Reverberation during Slow-Wave Sleep in Multiple Forebrain Areas

**DOI:** 10.1371/journal.pbio.0020024

**Published:** 2004-01-20

**Authors:** Sidarta Ribeiro, Damien Gervasoni, Ernesto S Soares, Yi Zhou, Shih-Chieh Lin, Janaina Pantoja, Michael Lavine, Miguel A. L Nicolelis

**Affiliations:** **1**Department of Neurobiology, Duke University Medical CenterDurham, North CarolinaUnited States of America; **2**Institute of Statistics and Decision Sciences, Duke UniversityDurham, North CarolinaUnited States of America; **3**Department of Biomedical Engineering, Duke UniversityDurham, North CarolinaUnited States of America; **4**Department of Psychological Brain Sciences, Duke UniversityDurham, North CarolinaUnited States of America; **5**Duke University Center for Neuro-Engineering, Duke UniversityDurham, North CarolinaUnited States of America

## Abstract

The discovery of experience-dependent brain reactivation during both slow-wave (SW) and rapid eye-movement (REM) sleep led to the notion that the consolidation of recently acquired memory traces requires neural replay during sleep. To date, however, several observations continue to undermine this hypothesis. To address some of these objections, we investigated the effects of a transient novel experience on the long-term evolution of ongoing neuronal activity in the rat forebrain. We observed that spatiotemporal patterns of neuronal ensemble activity originally produced by the tactile exploration of novel objects recurred for up to 48 h in the cerebral cortex, hippocampus, putamen, and thalamus. This novelty-induced recurrence was characterized by low but significant correlations values. Nearly identical results were found for neuronal activity sampled when animals were moving between objects without touching them. In contrast, negligible recurrence was observed for neuronal patterns obtained when animals explored a familiar environment. While the reverberation of past patterns of neuronal activity was strongest during SW sleep, waking was correlated with a decrease of neuronal reverberation. REM sleep showed more variable results across animals. In contrast with data from hippocampal place cells, we found no evidence of time compression or expansion of neuronal reverberation in any of the sampled forebrain areas. Our results indicate that persistent experience-dependent neuronal reverberation is a general property of multiple forebrain structures. It does not consist of an exact replay of previous activity, but instead it defines a mild and consistent bias towards salient neural ensemble firing patterns. These results are compatible with a slow and progressive process of memory consolidation, reflecting novelty-related neuronal ensemble relationships that seem to be context- rather than stimulus-specific. Based on our current and previous results, we propose that the two major phases of sleep play distinct and complementary roles in memory consolidation: pretranscriptional recall during SW sleep and transcriptional storage during REM sleep.

## Abstract 2

Rats exposed to novel objects during periods of wakefulness generate neural activity that is correlated with patterns observed in subsequent sleep episodes

## Introduction

Sleep is important for the consolidation of newly acquired memories (Jenkins and Dallenbach 1924; Fishbein 1971; Pearlman and Becker 1974; Smith and Butler 1982; Smith and Kelly 1988; Karni et al. 1994; Stickgold et al. 2000; Laureys et al. 2002; Fenn et al. 2003). The discovery of experience-dependent neuronal reactivation during sleep (Pavlides and Winson 1989) corroborated the notion that novel memory traces, after successful encoding, must be replayed in their supporting neuronal networks until synaptic plasticity can effect trace consolidation (Hebb 1949; Gutwein et al. 1980; Winson 1985; Ribeiro et al. 1999). Postacquisition neuronal reactivation during sleep or quiet waking (WK) was found to preserve the temporal relationships of alert, exploratory WK in the hippocampus (HP) (Wilson and McNaughton 1994; Skaggs and McNaughton 1996; Nadasdy et al. 1999; Poe et al. 2000; Louie and Wilson 2001; Lee and Wilson 2002) and the cerebral cortex (CX) (Qin et al. 1997; Hoffman and McNaughton 2002), causing a correlated replay of activity patterns across two-neuron (Wilson and McNaughton 1994) or many-neuron (Louie and Wilson 2001) ensembles. To date, experience-dependent brain reactivation during sleep has been observed in rodents (Pavlides and Winson 1989; Wilson and McNaughton 1994; Skaggs and McNaughton 1996; Qin et al. 1997; Nadasdy et al. 1999; Louie and Wilson 2001; Lee and Wilson 2002), nonhuman primates (Hoffman and McNaughton 2002), humans (Maquet et al. 2000), and even songbirds (Dave and Margoliash 2000), pointing to a very general biological phenomenon. Importantly, postacquisition brain reactivation during sleep has been shown to be proportional to memory acquisition in rats (Gerrard 2002) and humans (Peigneux et al. 2003).

In spite of the positive evidence, the brain reactivation hypothesis for memory consolidation during sleep faces several objections. First, the neocortical reactivation detected to date is extremely subtle and decays rapidly within less than 1 h of memory trace formation (Qin et al. 1997; Hoffman and McNaughton 2002). Such transient reactivation falls short of explaining the disruption of memory traces by sleep deprivation several hours and even days after initial acquisition (Fishbein 1971; Pearlman and Becker 1974; Smith and Butler 1982; Smith and Kelly 1988; Karni et al. 1994; Stickgold et al. 2000; Fenn et al. 2003). Second, *strictu sensu* neuronal reactivation during sleep in mammals has only been investigated in the hippocampocortical loop (Pavlides and Winson 1989; Wilson and McNaughton 1994; Skaggs and McNaughton 1996; Qin et al. 1997; Nadasdy et al. 1999; Louie and Wilson 2001; Hoffman and McNaughton 2002; Lee and Wilson 2002), making it difficult to ascertain whether the phenomenon is particular to this neural circuit or whether it represents global experience-dependent changes in the brain. Third, brain reactivation has mostly been observed in highly trained animal subjects (Wilson and McNaughton 1994; Skaggs and McNaughton 1996; Qin et al. 1997; Nadasdy et al. 1999; Dave and Margoliash 2000; Louie and Wilson 2001; Hoffman and McNaughton 2002; Lee and Wilson 2002), raising skepticism about its relevance for the acquisition and consolidation of novel information (Kudrimoti et al. 1999). Finally, experience-dependent brain reactivation has been reported to occur in all behavioral states (Pavlides and Winson 1989; Wilson and McNaughton 1994; Skaggs and McNaughton 1996; Qin et al. 1997; Louie and Wilson 2001; Lee and Wilson 2002), including WK (Nadasdy et al. 1999; Hoffman and McNaughton 2002). Although the first finding in this regard has hinted at a possible predominance of reactivation during slow-wave (SW) sleep (Pavlides and Winson 1989), a comprehensive comparison of the relative contributions of WK, SW, and rapid eye-movement (REM) sleep for brain reactivation is still missing. To further complicate the issue, recent studies have raised the possibility that neuronal processing may occur at either slower or faster speed than normal physiological rates during REM (Louie and Wilson 2001) and SW (Nadasdy et al. 1999; Lee and Wilson 2002) sleep, respectively. Thus, it is uncertain at the moment how brain reactivation relates to different behavioral states.

**Figure 1:**
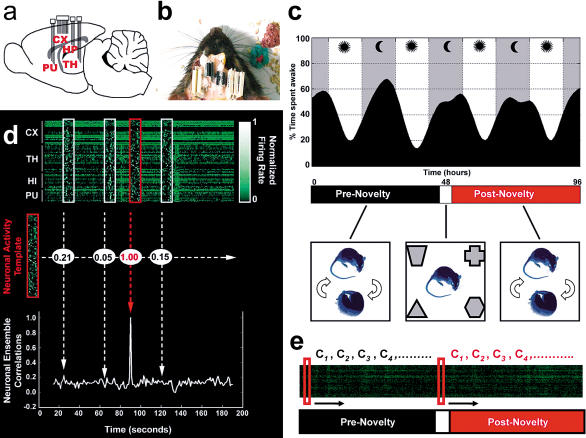
Methodology (A) Neuroanatomical location of multielectrode implants, indicated on a schematic parasaggital section based on Paxinos and Watson (1997). Indicated are the cerebral cortex (CX), the hippocampus (HP), the thalamus (TH), and the putamen (PU). (B) Top view of a rat implanted with several multielectrode arrays. (C) Experimental design. The upper panel shows a representative example of the strong circadian dynamics of the rat sleep–wake cycle (rat 5). Gray bands indicate lights-off; white bands indicate lights-on. Notice the fixed 12-h periods of darkness and light. The lower panels show animals continuously recorded for up to 96 h that were kept undisturbed except for a 1-h period of novel CSS (white segment) produced by the tactile exploration of four distinct novel objects placed at the corners of the recording box. Neural data from pre- and postnovelty periods (black and red segments, respectively, in the middle panel) were compared. (D) Neuronal ensemble correlation method. Neuronal activity templates (red boxes) were compared with extensive recordings of neuronal action potentials (green ticks in upper panel) by way of an offline template-matching algorithm (Louie and Wilson 2001) that generalizes the notion of pairwise correlations to neuronal ensembles of any size. Templates and targets (white boxes) were binned, firing-rate normalized, and correlated (middle panel). This procedure yields a time series of neuronal ensemble correlations for each template–target sliding match (lower panel). (E) Templates of interest (red boxes) were sampled around the origin of pre- and postnovelty periods during alert WK and slid against their corresponding neuronal targets so as to sample neuronal correlations every 30 s for up to 48 h.

In order to address these objections, we set out to investigate the effects of a transient novel tactile experience on the long-term evolution of ongoing brain activity across the major behavioral states of the rat. In each of the five animals studied, extracellular activity of 59–159 neurons per animal and local field potentials (LFPs) representing larger-scale neural rhythms were simultaneously recorded from four different brain regions: HP, primary somatosensory “barrel field” CX, ventral posteromedial thalamic nucleus (TH), and putamen (PU) (Figure 1A and 1B; see also Figures S6 and S7). These brain regions were chosen because they comprise three major forebrain circuit loops essential for rodent species-specific behaviors. Rats are nocturnal gatherers that exhibit a variety of exploratory behaviors during the night, sleeping intermittently and mostly during the day (Timo-Iaria et al. 1970) (upper panel in Figure 1C). In the wild, rats rely on spatial navigation and superb whisker-based tactile discrimination to explore new territories in search of food (Nowak 1999). The corticothalamic, corticohippocampal, and corticostriatal loops probed in this study have been implicated in tactile information processing (Simons 1978; Ghazanfar et al. 2000), spatial navigation and memory formation (O'Keefe 1976; Squire 1992), and the execution of complex motor sequences (Dieckmann and Hasser 1968; Jog et al. 1999).

In our studies, neural signals were continuously recorded across the natural sleep–wake cycle for 48–96 h, with a single 1-h exposure to four complex objects placed in the four corners of the recording box (Figure 1C). All objects were strictly novel to the subjects and were designed to maximize shape, texture, and behavioral value differences (Figure S1). Objects were presented half-way through the recording time (01:16 a.m. ± 00:51, mean ± SD) when lights were off and WK peaked (see Figure 1C), so as to maximize the drive for whisker-based tactile exploration of the environment. The experiment, therefore, consisted of a naturalistic behavioral paradigm involving multiple novel sensory and spatial cues; it was designed to maximize changes induced by exposure to novel objects, as opposed to changes related to repeated behavioral training. As expected, this paradigm increased WK relative to sleep during the exposure time (Figure S2), leading to novel complex sensory stimulation (CSS). Other than novel stimulation and the periodic removal of waste and introduction of food pellets and water, animals were kept undisturbed in the same environment throughout the recordings. Our paradigm produced marked and acute exploratory behavior (Figure S2) without disrupting the large-scale sleep–wake structure across the many hours of recording (see upper panel in Figure 1C).

In order to investigate the long-term effects of novel stimulation on the spatiotemporal evolution of ongoing neuronal activity, we took advantage of a neuronal ensemble correlation method previously shown to detect experience-dependent reactivation of rodent hippocampal ensembles during SW and REM sleep (Louie and Wilson 2001). This method generalizes the concept of pairwise neuronal correlations (Qin et al. 1997; Hoffman and McNaughton 2002) to an arbitrarily large number of neurons, quantifying the degree of similarity between spatiotemporal patterns of neuronal activity by way of a firing rate-normalized template-matching algorithm (see Figure 1D). Templates of alert WK neuronal ensemble activity were selected from moments when animals made whisker contact with the novel objects (*n*= five templates per animal). Control templates were selected from epochs of alert WK 24 h or 48 h before novel stimulation (24 h for rats 1–3, 48 h for rats 4 and 5; *n* = five templates per animal), during which familiar tactile stimulation was produced by the contact of whiskers with the smooth walls of the recording box, to which animals had been habituated. Templates were matched against the entire record of neuronal activity using the neuronal ensemble correlation method (see Figure 1E). The resulting correlation temporal profiles were averaged for each template set, aligned with reference to the light–darkness cycle to control for possible circadian effects, and compared.

## Results

### Novelty-Induced Patterns of Neuronal Activity Reverberate for up to 48 h

**Figure 2:**
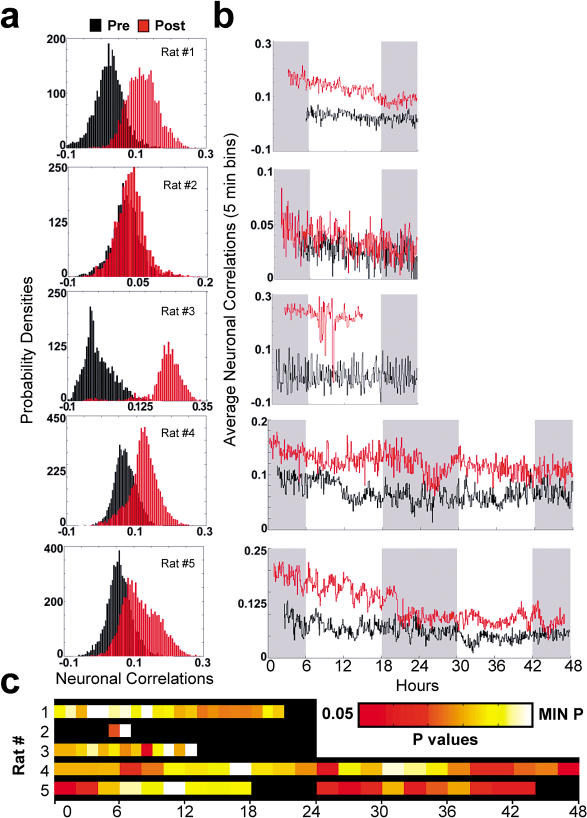
Neuronal Ensemble Correlations Including up to Four Forebrain Regions Reveal Long-Lasting Reverberation (A) Postnovelty neuronal correlations were significantly larger than prenovelty correlations in all animals studied. (B) Temporal profiles of neuronal ensemble correlations. Gray bands indicate lights-off; white bands indicate lights-on. (C) Temporal evolution of *p* values associated with pre- and postnovelty Bonferroni comparisons performed in intervals of 1 h (rats 1–3) or 2 h (rats 4 and 5). Significant experience-dependent neuronal reverberation was detected up to 48 h after novel stimulation. Color bar in linear scale; black denotes *p* > 0.05. The minimum *p* values (MIN P) were, respectively, 10^−14^, 10^−3^, 10^−12^, 10^−23^, and 10^−22^.

First, we tested whether the neuronal ensemble correlation method could detect any trace of neuronal reverberation lasting at least more than 1 h after exposure to novel stimulation. For this, we examined correlation profiles obtained for all recorded neurons (three to four brain areas pooled together) in each animal. As shown in Figure 2A, postnovelty average correlation distributions were significantly right-shifted relative to prenovelty distributions (ANOVA of mean pre- and postnovelty correlations over 24 h or 48 h; *n* = five animals, *F* = 9.5, *d.f.* 1, *p* = 0.016). This indicates that the neuronal firing patterns concomitant with novel stimulation persisted significantly more during the ensuing time than patterns sampled 24 h or 48 h before novel stimulation, when animals were in the same behavioral state (alert WK), but without novel objects to explore. The effect was independently observed, to a variable degree, in all the five animals studied (Bonferroni comparison, *p* < 0.01). Figure 2B shows the temporal evolution of neuronal ensemble correlations for 24 h (upper three panels in Figure 2B) and 48 h (lower two panels in Figure 2B).

Despite the marked interanimal variability in the shapes and magnitudes of these profiles, a significant increase of neuronal correlations after exposure to novel stimulation was observed in most recording sites. Importantly, these increases lasted well above 1 h, as revealed by the temporal evolution of the *p* values generated by Bonferroni comparisons between post- and prenovelty correlation distributions (Figure 2C). These results indicate that significant experience-dependent neuronal reverberation could be detected in the forebrain up to 48 h after exposure to novel stimulation.

In order to assess the contributions of different neurons to total ensemble correlations, we ran the correlation analysis on a neuron-by-neuron basis. We found that no one subset of neurons was particularly responsible for the reverberation effect, since the contribution of individual neurons was highly variable in time (data not shown). This indicates that the neuronal changes associated with exposure to novel stimuli were highly distributed through the neuronal populations sampled. Furthermore, judging by the maximum neuronal ensemble correlations observed (rat 3, peak at 0.35; Figure 2B), one would conclude that novel stimulation-specific neuronal activity was not perfectly repeated, but was rather loosely reverberated for several hours.

### Neuronal Reverberation Occurs in Multiple Forebrain Areas

**Figure 3:**
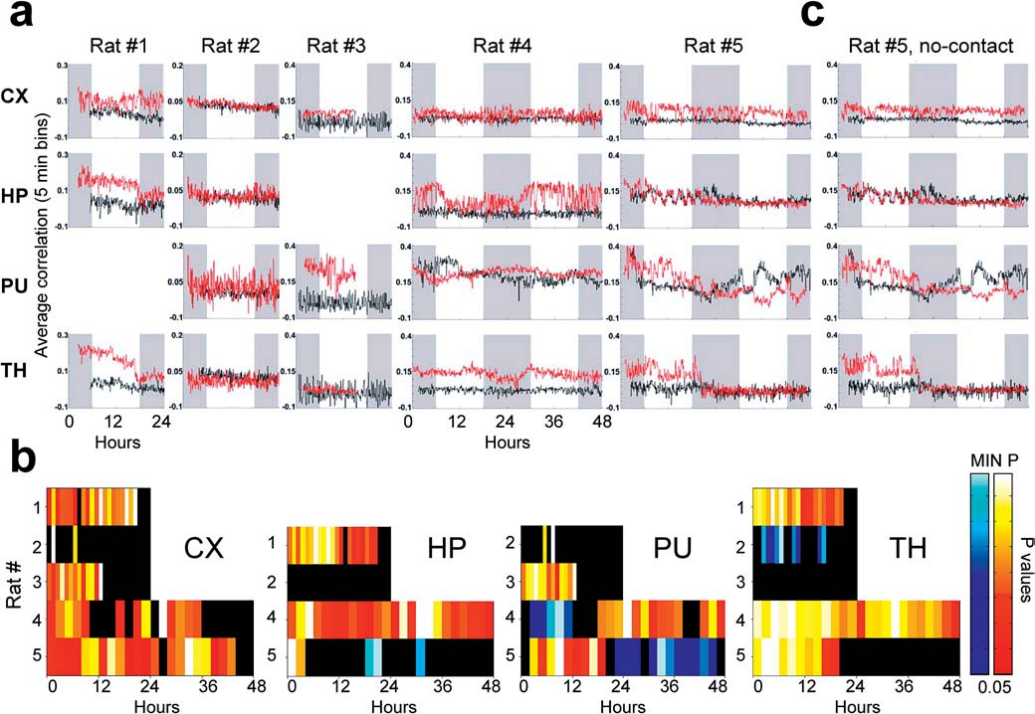
Long-Lasting Neuronal Reverberation Occurs in the CX, HP, PU, and TH (A) Temporal profiles of neuronal ensemble correlations in all recording sites. Gray bands indicate lights-off; white bands indicate lights-on. Red and black traces indicate post- and prenovelty correlations, respectively. (B) Temporal evolution of *p* values associated with pre- and postnovelty Bonferroni comparisons for individual brain areas, calculated as in Figure 2C. Color bar in linear scale; black denotes *p* > 0.05. Minimum *p* values (MIN P) in crescent “rat number” order, as follows: CX: 10^−10^, 10^−4^, 10^−5^, 10^−9^, 10^−9^; HP: 10^−12^, not significant, 10^−22^, 10^−3^ (both red and blue scales); PU: 10^−2^, 10^−12^, 10^−15^ (blue scale) and 10^−17^ (red scale), 10^−16^ (red scale) and 10^−34^ (blue scale); TH: 10^−18^, 10^−3^ (blue scale), not significant, 10^−30^, 10^−16^. (C) Neuronal ensemble correlations for no-contact templates (taken from epochs within the novel stimulation period in which animals had no sensory contact with the novel objects) also show enhanced neuronal reverberation.

In order to assess the anatomical distribution of experience-dependent neuronal reverberation, we performed the neuronal ensemble correlation analysis for each area separately (Figure 3A). At first glance, differences between pre- and postnovelty correlation profiles were noticeable in all animals, with predominant effects in a different subset of areas for each animal. For example, rat 4 showed marked reverberation in the HP, but small changes in the CX, while rat 5 showed just the opposite. In the majority of the recorded sites, postnovelty traces (red in Figure 3A) run above prenovelty traces (black in Figure 3A), but the reverse also occurs, suggesting some sort of antireverberation. The most widespread reverberation was seen in rat 1, which showed sustained reverberation in the CX and decaying reverberation in the HP and TH. A somewhat similar pattern was seen in rat 5, while rat 3 showed strong reverberation only in the PU, and rat 4 in the HP and TH. Rat 2 showed the least reverberation of all, with somewhat stronger effects in the PU.

Despite the interanimal and interarea differences in the magnitude and shape of correlation profiles, significant changes between pre- and postnovelty correlations were observed in all areas studied (CX, five of five rats; HP, three of four rats; PU, four of four rats; and TH, four of five rats; Bonferroni comparison, *p* < 0.05). Indeed, experience-dependent changes were not statistically different across different forebrain areas (ANOVA, *F* = 0.24, *d.f.* 3, *p* = 0.86). The temporal evolution of *p* values (Bonferroni comparison) associated with single-area correlation profiles shows that significant reverberation was present in 16 of 18 recording sites for several hours after exposure to novel stimulation (Figure 3B). It also confirms that neuronal ensemble reverberation (post-/prenovelty correlations) is not the only kind of experience-dependent change possible. Some animals showed significant long-lasting antireverberation (pre-/postnovelty correlations), i.e., patterns of activity that were statistically more dissimilar from novel stimulation templates than expected by chance. Antireverberation (indicated by blue hues in Figure 3B) occurred in the HP (one of four rats), PU (two of four rats), and TH (one of five rats), but not in the CX. Single-area postnovelty average correlations showed peaks of the order of 0.4 (rat 5, PU; Figure 3A), but typically ranged from 0.1 to 0.2. Therefore, high-fidelity replay of neuronal firing patterns was not observed even when single areas were considered.

An intriguing observation came from the scrutiny of no-contact templates of neuronal activity, sampled within the novel stimulation 1-h period during alert WK, but excluding moments of contact between whiskers and objects. Surprisingly, no-contact templates yielded correlation profiles that were almost indistinguishable from those obtained when animals had tactile contact with the novel objects (Figure 3C). Therefore, the exploration of the novel environment enhanced the reverberation of all the neuronal activity patterns concomitant with the experience and not just of those corresponding to moments in which animals received sensory inputs from the objects. This rules out the possibility that stimulus complexity, rather than novelty, is the underlying cause of the enhanced neuronal reverberation observed after exploration of the objects.

### Neuronal Reverberation Peaks during SW Sleep

Single-area results indicate that neuronal ensemble correlations often peak during discrete epochs that last a few hours. We also noticed marked oscillations of the correlation trace in several recorded sites (e.g., rat 5, CX). These observations suggest that some underlying biological process, with slow evolution but with sharp phase transitions, governs the long-term reverberation of neuronal firing patterns. To test whether transitions in the wake–sleep cycle could amount for these effects, we investigated how experience-dependent changes in neuronal correlations varied across the three major rat behavioral states: WK, SW sleep, and REM sleep. A comparison across states of post-/prenovelty correlation ratios calculated from averages of entire recordings indicated a significant state-specific effect (ANOVA, *F* = 9.289, *d.f.* 2, *p*= 0.0004), with SW ratios being significantly higher than those of both WK (Bonferroni comparison, *p* < 0.05) and REM (Bonferroni comparison, *p* < 0.003). Indeed, significant state-specific differences in post-/prenovelty correlation ratios were individually detected in four of five animals (ANOVAs, *d.f.* 2: rat 2, PU, *F* = 4.13, *p* = 0.039; rat 3, CX, *F* = 6.45, *p* = 0.026; rat 4, HP, *F* = 3.99, *p* = 0.029; rat 5, CX, *F* = 13.81, *p* < 0.0001).

**Figure 4:**
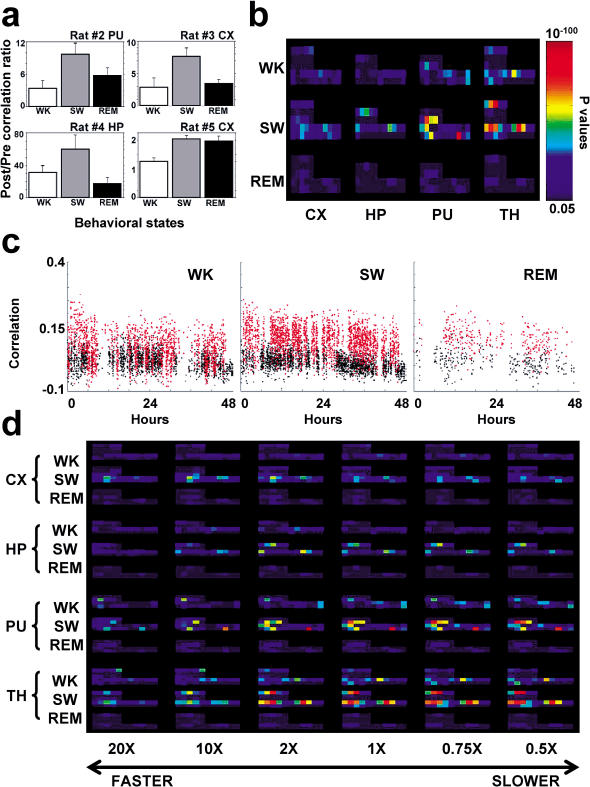
Neuronal Reverberation Depends on Behavioral State (A) Histograms (mean *±* SEM) of post- and prenovelty correlation ratios in the recording sites where significant state-related differences in neuronal ensemble correlations were detected. SW sleep post-/prenovelty correlation ratios were significantly higher than WK in all four cases (Bonferroni comparison *p* values as follows: rat 2, PU, SW>WK 0.013; rat 3, CX, SW>REM 0.017 and SW>WK 0.022; rat 4, HP, SW>REM 0.013 and SW>WK 0.039; rat 5, CX, SW>WK 0.0001 and REM>WK 0.0002). (B) Bonferroni comparison *p* values for post-/prenovelty comparisons in all animals according to behavioral state and brain area, calculated in intervals of 4 h. Animal order and time as in Figure 3B. Color bar in linear scale. (C) Neuronal ensemble correlations sorted by state for rat 5 CX. In comparison with WK, there is a clear increase in the contrast between pre- and postnovelty correlations during SW sleep. In this particular animal and brain area, increased correlations were also seen for REM sleep, but this was not the case in other animals (A). Furthermore, this REM effect was substantially weakened when expressed in *p* values (B), due to the very short duration of REM sleep episodes. In this respect, notice that REM sleep has much fewer datapoints, reflecting the short duration of this state relative to WK and SW sleep. Thus, even in a site where REM sleep showed results similar to SW sleep, the cumulative neuronal reverberation that takes place during REM is necessarily less than that of SW. (D) Statistical comparison of matches between templates of neuronal activity sampled at WK normal speed with a range of targets spanning different temporal scales. Plotted are Bonferroni comparison *p* values for post- and prenovelty comparisons in all animals according to behavioral state and brain area, calculated in intervals of 4 h for speed factors ranging from 20 times faster to two times slower than the WK normal rate. No evidence for optimization at speeds different from the WK physiological rate (1×) was found. Color scale as in (B).

The mean correlation values found in those recording sites for the three behavioral states reveal that SW correlations were systematically larger than WK correlations (Figure 4A). Several other recorded sites displayed similar but nonsignificant trends. Meanwhile, the REM correlations mea-sured were variable and could not be consistently ranked in relation to WK and SW sleep. Comparable neuronal reverberation between SW and REM sleep was observed in only one animal (rat 5, CX). A major effect of SW sleep on neuronal reverberation was corroborated by the temporal evolution of successive state-specific Bonferroni comparison *p* values calculated for pre- and postnovelty 4-h average correlations across all animals and brain areas studied (Figure 4B). The strongest contrast between pre- and postnovelty neuronal correlations was clearly seen during SW sleep, with less effect seen in WK and even less in REM sleep. Figure 4C depicts the state-sorted pre- and postnovelty correlations for rat 5, CX, illustrating both the general SW effect and the much less-prevalent REM sleep changes.

Next, we tested the possibility reported in hippocampal place cells (Nadasdy et al. 1999; Louie and Wilson 2001; Lee and Wilson 2002) that experience-dependent replay of neuronal firing patterns during sleep can be slower (REM) or faster (SW) than during WK. Template-to-target matches at different speed factors were obtained by comparing 250 ms-binned templates with targets binned within a range of different bin sizes (from 12.5 ms to 500 ms). By temporally compressing and expanding “target” spike records before matching them to templates, we determined the magnitude of neuronal ensemble correlations for speed factors ranging from 0.5 to 20 times the physiological WK processing speed, which covers the reported optimum speed ranges for SW (Lee and Wilson 2002) and REM sleep (Louie and Wilson 2001). A predominance of neuronal reverberation during SW sleep was seen for all speed factors, as indicated by Bonferroni comparisons (Figure 4D). However, no significant differences were seen when post- and prenovelty correlation ratios (calculated from averages of entire recordings) were compared across different speed factors (ANOVA, *F* = 1.496, *d.f.* 5, *p* = 0.19). Figure 4D shows that within any given state or area, neuronal reverberation did not vary systematically with speed factor, and the temporal distribution of correlation hot-and-cold spots was largely insensitive to speed factor.

Thus, we found no evidence that forebrain neuronal reverberation can be optimized assuming replay speeds different from the WK normal rate. Indeed, a subtle but consistent decrease of *p* values can be observed for speed factors 10 times and 20 times faster than normal WK rates, while speed factors near the physiological range (2×–0.5×) show stronger and similar effects. This was the case even in the HP, in contrast with previous findings in hippocampal place cells recorded in highly trained animals performing a spatial navigation task (Nadasdy et al. 1999; Louie and Wilson 2001; Lee and Wilson 2002). At present, it is unclear whether this discrepancy reflects differences in stimulus familiarity (novel versus habitual), stimulation modality (tactile exploration versus spatial navigation), the very low representation of place cells in our hippocampal samples (less than 5%), or possible differences in the analyses used in previous studies, based on the statistical boot-strapping of relatively small datasets (Nadasdy et al. 1999; Louie and Wilson 2001; Lee and Wilson 2002).

**Figure 5:**
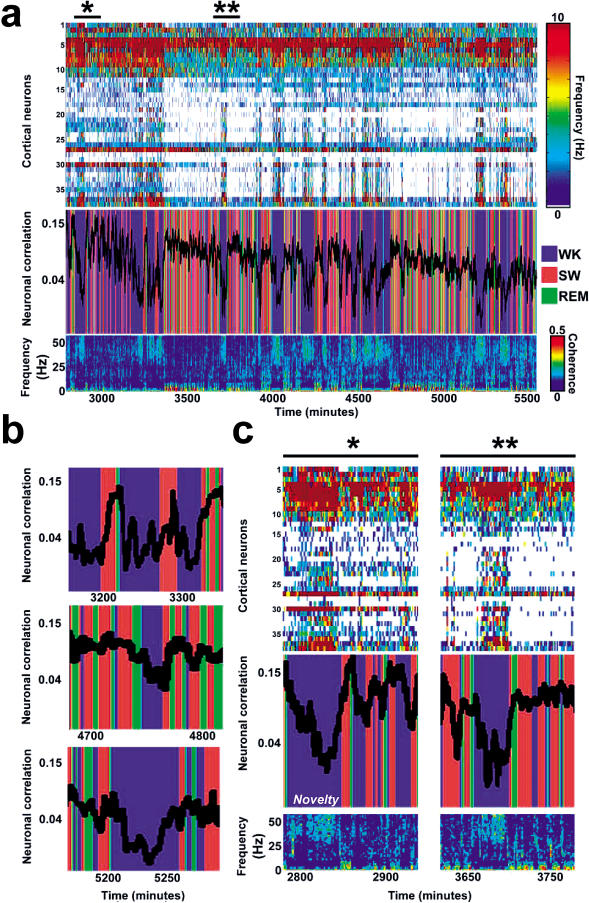
Neuronal Reverberation Is Strongest during SW Sleep (A) Rat 5 (CX) dramatically illustrates the state dependency of neuronal ensemble correlations, which are strongly increased by SW sleep but readily decreased by WK. The upper panel shows the firing rates of 38 cortical neurons for approximately 45 h after exposure to novel stimulation (indicated by an asterisk). The middle panel shows the superimposition of successive neuronal ensemble correlations and concurrent behavioral states. Nearly all correlation peaks correspond to SW episodes, while almost all troughs match WK epochs. The lower panel represents pooled LFP forebrain coherence (Amjad et al. 1997) over time, useful to discriminate between WK (strong coherence above 25 Hz and weak coherence under 5 Hz) and SW sleep (the opposite). Notice that in this particular example (rat 5, CX), REM episodes show correlations similar to those of SW sleep, but, as shown in Figure 4, this was the exception and not the rule across several animals. (B) State-dependent neuronal reverberation was sustained throughout the recording period, as shown by segments representing the beginning (3,200–3,300 min), middle (4,700–4,800 min), and end (5,200–5,250 min) of the experimental record. In the upper panel, notice the progressive increase of neuronal correlations across single SW sleep episodes. (C) Blow-up of two selected data segments indicated by asterisks in (A). Despite having being sampled from moments of high neuronal firing rates (asterisk), novel stimulation templates reverberate most strongly during SW sleep when firing rates are low (single asterisk and double asterisks). The high firing rates that characterize WK correspond to decreased neuronal reverberation, probably due to sensory interference.

All together, our results indicate that neuronal reverberation was consistently stronger during SW sleep, decreasing during WK. This is remarkably well-illustrated by a superimposition of behavioral state classification and neuronal ensemble correlations (middle panel in Figure 5A), which reveals an exquisite long-term temporal match between epochs of increased neuronal correlations and SW episodes (red in Figure 5A). Likewise, neuronal correlation troughs show a tight correspondence with WK episodes (blue in Figure 5A). This characteristic state-dependency persisted throughout the 45 h of postnovelty recording (see Figure 4B). Notice that REM sleep only showed SW-like results in one out of five animals (rat 5, CX; depicted in Figures 4 and 5). In the remaining animals, REM correlations were either closer to WK levels than to SW levels or in between (see Figure 4A). Given this marked variability and the very short duration of total REM sleep in comparison with total SW sleep (WK 52%, SW 40%, and REM 8% of total recording time for five animals), this indicates that REM sleep plays a minor role in neuronal reverberation.

Interestingly, a comparison of the correlation temporal profile with the concurrent neuronal firing record (upper panel in Figure 5A) reveals that SW correlation peaks correspond to periods of decreased firing rate, while WK correlation troughs match epochs of increased neuronal activity. This is better shown in Figure 5C, which depicts data segments approximately 2-h long, comprising the three major behavioral states studied. The first segment (shown by a single asterisk in Figure 5A) corresponds to 60 min of novel stimulation and the immediately ensuing sleep–wake cycles, while the second segment (shown by double asterisks in Figure 5A) illustrates sleep–wake episodes occurring approximately 15 h after the original experience. Thus, although novel stimulation templates of neuronal activity were selected from WK episodes characterized by high firing rates, ensuing reverberation of these neuronal firing patterns was most pronounced during SW sleep, under lower firing rates.

## Discussion

In order to assess several objections to the replay hypothesis for memory consolidation during sleep, we conducted long-term continuous neuronal recordings on animals subjected to a naturalistic behavioral paradigm, which involved multiple novel sensory and spatial cues. Our results indicate that large-scale neuronal firing patterns generated during the exploration of novel objects can recur for several hours after the reference experience throughout most of the forebrain, while firing patterns associated with familiar stimulation (i.e., the walls of the recording box) are substantially less detectable over time.

Our results fend off three major objections to the notion that neuronal reverberation during sleep may underlie memory consolidation. First, significant experience-depen-dent changes in neuronal ensemble correlations can be tracked as late as 48 h after the reference novel experience, being therefore compatible with memory impairment effects of sleep deprivation applied hours or days after training (Fishbein 1971; Pearlman and Becker 1974; Smith and Butler 1982; Smith and Kelly 1988; Karni et al. 1994; Stickgold et al. 2000; Fenn et al. 2003). Second, these effects were observed in rats completely naïve with respect to the reference stimuli, ruling out the possibility that only the performance of highly trained behaviors would be followed by neuronal reverberation. Third, neuronal ensemble correlations were significantly enhanced during SW sleep and decreased during WK, while REM sleep produced variable results. The data indicate that novel experience caused sustained neuronal reverberation (Hebb 1949) rather than discrete reactivation (Wilson and McNaughton 1994; Kudrimoti et al. 1999), in the sense that reverberation decreased, but did not disappear, during WK (see Figure 4C). The consistent increase in neuronal reverberation during SW sleep, the high interanimal variability of neuronal reverberation during REM sleep, and the small contribution of REM sleep to total sleep time indicate that the cognitive effects of experience-dependent neuronal reverberation (Gerrard 2002; Peigneux et al. 2003) must be largely attributed to SW sleep. Therefore, our results suggest a major role for SW sleep in the reverberation of new memory traces.

An important result of the present study is the inverse correlation between neuronal correlations and concurrent firing rates. Although all neuronal activity templates were taken from epochs of high arousal WK when firing rates were generally high, their neuronal reverberation during subsequent WK was not very prominent (see Figures 4 and 5). In contrast, reverberation of the same activity templates peaked during SW sleep, when the firing rates of forebrain neurons are generally low (see Figures 4 and 5). This suggests that reverberating patterns of neuronal activity associated with past novel experience are largely—but not completely—masked during WK by incoming sensory inputs unrelated to the reference experience. By the same token, peak neuronal correlations arise during SW sleep, when sensory interference ceases. Taken together, these observations corroborate the notion that the importance of sleep for memory consolidation stems from the offline processing of memory traces, i.e., from the absence of sensory interference (Jenkins and Dallenbach 1924; Melton and Irwin 1940; Winson 1985). Were there differences in average firing rate before and after exposure to the novel environment, and could such differences account for the effects seen in neuronal correlations? The neuronal ensemble correlation method (Louie and Wilson 2001) involves a normalization of firing rates after binning, and therefore it is insensitive to moderate changes in the mean firing rate. In our experiment, firing rates for individual neurons varied substantially during exposure to novelty, with some neurons firing more and other neurons firing less than before exposure. This variability caused a moderate but nonsignificant increase in the average activity of the cells so that the firing rates within novelty templates were on average approximately 10% higher than the neuronal firing rates of preexposure templates. Neuronal firing rates increased during exposure to novel objects and persisted elevated for up to 1 h after removal of the objects, returning to baseline afterwards. This contrasts with the timecourse of neuronal correlations changes, which were increased for up to 48 h. Finally, as explained above, neuronal reverberation was inversely correlated with firing rates. Thus, mean firing-rate differences were not responsible for the reverberation effect, which should rather be attributed to specific firing-rate relationships across multiple neurons.

Our results impose some clear constraints on future sleep and learning theories.

First, no sign of neuroanatomical specificity was found in the correlations measured, and in particular no significant differences between hippocampal and extrahippocampal areas could be detected. Despite considerable interanimal variability in the magnitude of the correlations observed in the different brain structures, statistically significant neuronal reverberation produced by novel stimulation was observed in 16 out of 18 recorded brain sites, comprising the CX, HP, PU, and TH. This broad forebrain reverberation was related to the free exploration of four novel and complex objects, placed in four well-separated places and including the presence of novel food. Thus, novel experience involving tactile, gustatory, olfactory, spatial, and motor components is able to engage multiple forebrain structures, all similarly capable of reverberating neuronal patterns of activity after novel stimulation.

Second, neuronal ensemble correlations measured across the forebrain were typically small (on the order of 0.1–0.3), agreeing with values previously reported for pairwise (Wilson and McNaughton 1994; Skaggs and McNaughton 1996; Qin et al. 1997; Hoffman and McNaughton 2002) or many-neuron (Louie and Wilson 2001) correlations. Qualitatively similar results were observed for bins ranging from 5 ms to 1,000 ms, with higher correlation values for larger bin sizes. This suggests that neurons of multiple forebrain areas, once exposed to novel experience, do not accurately replay prior WK activity patterns longer than 5 ms. Instead, they show a mild but long-lasting bias towards (or against) the reference activity patterns. Indeed, not a single template-to-target match (out of 979,200 matches sampled) yielded correlation values higher than 0.45, indicating that novelty-induced neuronal reverberation occurs at low fidelity. It has been proposed that a high-fidelity replay of neuronal firing patterns during sleep may be achieved assuming that replayed patterns can undergo time compression and expansion (Nadasdy et al. 1999; Louie and Wilson 2001; Lee and Wilson 2002). We assessed this possibility thoroughly, but found no evidence of such effects in any of the forebrain sites recorded. Thus, in the face of consistently low neuronal correlation values, the “high-fidelity replay hypothesis” for timeperiods larger than 5 ms should be rejected, at least in mammals (Dave and Margoliash 2000). It remains to be seen whether more precise patterns of spike-to-spike correlations may reverberate in intervals smaller than 5 ms.

A third important point regards the observation that neural activity sampled when animals were aroused, but not touching the objects, yielded neuronal reverberation that was nearly identical to that obtained when animals made sensory contact with the objects. This indicates that the kind of experience-dependent neuronal reverberation detected by the neuronal ensemble correlation method (Louie and Wilson 2001) does not reflect the specific features of the stimuli, but is related to the overall behavioral salience of the novel stimulation period, i.e., is context- rather than stimulus-specific. In principle, these results are compatible with a slow and progressive process of memory consolidation (Bryson and Schacher 1969), proportional to the novelty of the experience, and able to bind together a multitude of contextual cues related to its core sensory elements (Kohler 1947).

It has been suggested that the neuronal reverberation of newly acquired synaptic changes during SW sleep may lead to the recall and storage of new memories by way of “calcium-mediated intracellular cascades” capable of opening the “molecular gates to plasticity” (Sejnowski and Destexhe 2000). This hypothesis is partially contradicted by evidence that calcium-dependent gene expression related to synaptic plasticity is upregulated during REM sleep (Ribeiro et al. 1999, 2002), but not during SW sleep (Pompeiano et al. 1994). The present findings and the current literature suggest instead that SW and REM sleep play separate roles on memory consolidation, with memory recall occurring during SW sleep and memory storage taking place during REM sleep. According to this view, the deleterious effects of sleep deprivation on memory consolidation would be a consequence of the disruption of the underlying neuronal reverberation and gene expression during SW and REM sleep, respectively.

**Figure 6:**
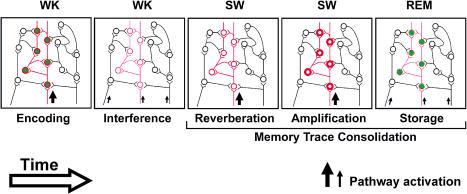
Conceptual Model of the Role of Sleep for Memory Consolidation Arrows indicate pathway activation by sensory inputs during WK, as well as intrinsic brain activity such as pontine waves during sleep (Datta 2000); different arrow sizes indicate different magnitudes of pathway activation. Red indicates calcium-dependent pretranscriptional processes, with different hue intensities representing the progressive amplification of recently acquired synaptic changes. Green indicates plasticity-related transcriptional regulation. The initial state of the model (data not shown) consists of environmental habituation, during which ongoing activity patterns only repeat themselves by chance. (First panel) A novel WK experience encodes a memory trace across multiple forebrain areas, selectively activating functionally related synapses. This triggers calcium-dependent pretranscriptional cascades (red) and plasticity-related gene expression (green) that lead to the common strengthening of the activated synapses. (Second panel) The continuation of WK involves a succession of unrelated sensory experiences capable of producing interference, i.e., a progressive weakening of recently encoded synaptic changes. (Third panel) Upon entering SW sleep, intrinsic brain activation is biased towards previously potentiated synapses, causing neuronal firing patterns originally produced during the novel WK experience to reverberate significantly above chance levels. (Fourth panel) The periodic activation of calcium-dependent second-messenger cascades by large-amplitude SW oscillations may result in the progressive amplification of the synaptic changes that encode the novel memory trace. (Fifth panel) SW-amplified synaptic changes are stored during REM sleep by way of plasticity-related transcriptional regulation.

The fact that neuronal reverberation is sustained for long epochs during SW sleep suggests that unconsolidated synaptic changes may not only be recalled, but also amplified over time during SW sleep. Indeed, a progressive increase of neuronal correlations across single SW sleep episodes was often observed (upper panel in Figure 5B). A model of how such amplification may arise is presented in Figure 6. We have recently proposed that the cyclical reiteration of trace amplification during SW sleep and trace storage during REM sleep promotes the postsynaptic propagation of memory traces (Pavlides and Ribeiro 2003), as suggested by the hippocampofugal pattern of gene expression during REM sleep (Ribeiro et al. 2002). Potentially, this propagation could cause memory traces to progressively reach farther and farther away from the original synaptic trajectory activated at initial encoding. Over time, this sleep-dependent propagation could lead to deeper encoding within the CX (Craik and Lockhart 1972; Cermak and Craik 1979), as well as hippocampal disengagement (Scoville and Milner 1957; Mishkin 1978; Kesner and Novak 1982; Squire 1992; Izquierdo and Medina 1997; Bontempi et al. 1999). The notion that the two major phases of sleep play distinct and complementary roles in memory consolidation is in line with evidence that SW and REM sleep have synergistic effects on human procedural learning (Mednick et al. 2003). Put in historical perspective, our model argues that sleep separately harbors both mechanisms postulated in the past (Hebb 1949) to be necessary for memory consolidation: postacquisition neuronal reverberation and structural synaptic plasticity. In conclusion, sustained neuronal reverberation during SW sleep, immediately followed by plasticity-related gene expression during REM sleep, may be sufficient to explain the beneficial role of sleep on the consolidation of new memories.

## Materials and Methods

### 

#### Chronic neuronal recordings

Multielectrode arrays (Figure S3) were surgically implanted according to National Institutes of Health (NIH) guidelines in five adult male Long–Evans rats (250–300 g). The following coordinates in millimeters relative to Bregma (Paxinos and Watson 1997) were used to center the arrays: HP (+2.8 anteroposterior [AP], +1.5 mediolateral [ML], −3.3 dorsoventral [DV]), CX (+3.0 AP, +5.5 ML, −1.5 DV), PU (−1.0 AP, +2.5 ML, −5.0 DV), and TH (+3.0 AP, +3.0 ML, −5.0 DV). Hippocampal data pool together neurons recorded with staggered electrodes from the CA1 field and the dentate gyrus. Locations of implants were histologically verified by comparing cresyl-stained frontal brain sections with reference anatomical planes (Paxinos and Watson 1997) (Figure S4). A Multineuron Acquisition Processor (128 channels; Plexon Inc., Dallas, Texas, United States) was used to perform recordings of neuronal spikes and LFPs, as previously described (Nicolelis et al. 1999) (Figures S5 and S6). A waveform-tracking technique involving periodic template adjustment was employed for the continuous recording of individual units (see Figure S5). Units that showed nonstationary waveforms, unstable firing-rate profiles, or both were discarded. The stability of firing rates within each behavioral state can be appreciated in the upper panel in Figure 5.

#### Behavior

Before the beginning of the experiment, animals were individually habituated to an empty recording box for 5–7 entire days (12 h:12 h light:dark schedule, lights on at 06:00 a.m.) so as to reach steady-state behavioral activity and baseline wake–sleep cycles. Behaviors were continuously recorded by way of two infrared-sensitive CCD video-cameras; infrared illumination was used to monitor behavior when visible lights were off. Behavioral states were coded as WK, SW sleep, or REM sleep based on a spectral analysis of LFPs and visual inspection of videotaped behaviors, according to previously described criteria (Ribeiro et al. 1999) (Figure S7).

#### Data analysis

Data were processed and analyzed by custom-made MATLAB^TM^ (MathWorks, Natick, Massachusetts, United States) code running in a computer cluster comprising 32 CPU (Evolocity^TM^, LNXI, Sandy, Utah, United States). Neuronal ensemble correlations were calculated following the method in Louie and Wilson (2001). In brief, a stretch of data (target) is scanned for similarity to certain multineuron temporal patterns of activity (templates). Templates consist of *CxN* matrices corresponding to simultaneously recorded spike trains of *C* neurons binned into *N* intervals of a certain length. Our results were obtained using 9 s-long templates binned with 250 ms-long bins with as many neurons as were available for the brain areas in question. Hence, each template pattern yielded a *Cx36* matrix. Target data comprising 24 h or 48 h were sparsely sampled (every 30 s) and binned into matrices of the same dimensions. For a *C*×*N* template data matrix *x* and a target data matrix *y* sampled at time *t*, the ensemble correlation index C_t_ is obtained by:



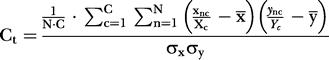



where



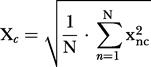


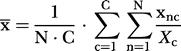


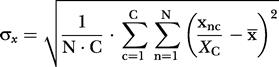



(the same applies to Y_c_, y¯, and σ_y_).

Templates were selected by careful scrutiny of behavior recorded on videotapes and of the corresponding spectral characteristics of hippocampal LFPs, so as to assure sampling during alert WK (Figure S7). Particular care was used to select template sets with a comparable prevalence of θ (5–8 Hz) over δ (2–4 Hz) frequencies (θ/δ hippocampal spectral ratios, ≥10). All templates and targets were individually sampled without overlap. Because binning may destroy higher-frequency phenomena, control analyses were run to assess the consequences of binning the data with bins of different sizes. Results obtained with bin sizes in the interval of 5–1,000 ms are qualitatively equivalent to the ones obtained using 250 ms bins (Figure S8). In order to detect temporally expanded or contracted repetitions of template patterns, a range of larger- or smaller-than-template bin sizes, respectively, was used when binning each target sample (12.5 ms, 25 ms, 125 ms, 250 ms, 375 ms, and 500 ms). This allowed us to compare targets spanning different temporal scales with templates of neuronal activity sampled at WK speed. The bin size range used allowed the detection of patterns temporally compressed by factors of 20, 10, and 2 (bin sizes of 12.5 ms, 25 ms, and 125 ms), temporally expanded by factors of 1.5 and 2 (bin sizes of 375 ms and 500 ms) as well as replayed at the same speed (250 ms). StatView^TM^ (SAS, Cary, North Carolina, United States) and MATLAB^TM^ software were used for descriptive statistics and hypothesis testing.

## Supporting Information

Figure S1 Objects Four different objects were used to produce CSS.
(9.7 MB PPT).

Figure S2 Behavior All animals were highly habituated to the recording box, so that exposure to novel complex objects caused a general increase in the animals' arousal. Four of five animals showed an increase in time spent in WK with respect to SW and REM sleep during CSS (A), as compared to adjacent pre- and postnovelty periods of equal length (60 min). The only exception was rat 1, which showed nevertheless a marked exploratory drive, spending nearly 20% of the exposure period in direct whisker-contact with the objects (B). Individual object preferences were moderately varied, as indicated in (C).
(746 KB PPT).

Figure S3 Multielectrode Arrays Teflon-coated tungsten wires (50 μm diameter, 300 μm between wires, 1.0–1.2 MΩ at 1 KHz; California Fine Wire Company, Grover Beach, California, United States) were assembled in multielectrode arrays shaped to fit different neuroanatomical targets.
(282 KB PPT).

Figure S4 Location of Implants Frontal brain sections stained for cresyl-violet were used to determine the sites of electrode placement. Electrode tracks, tissue scars, and reference electrolytic lesions performed a few days before sacrifice were used to delimit the implant sites, indicated in red in the figure below. Numbers on the right represent standard AP coordinates (Paxinos and Watson 1997) in millimeters from Bregma.
(6.3 MB PPT).

Figure S5 Neuronal Recordings To record neuronal activity, differentiated neural signal was preamplified (2,000×–32,000×) and digitized at 40 KHz. Up to four neuronal action potentials per recording channel were sorted online (SortClient 2002, Plexon Inc.) and validated by offline analysis (Offline Sorter 2.3, Plexon Inc.) according to the following cumulative criteria: voltage thresholds greater than two standard deviations of amplitude distributions; signal-to-noise ratio greater than 2.5 (as verified on the oscilloscope screen); less than 1% of interspike intervals smaller than 1.2 ms; and stereotypy of waveform shapes, as determined by a waveform template algorithm and principal component analysis. In order to continuously record individual neurons for up to 96 h, we used an adaptive algorithm (available on SortClient 2002, Plexon Inc.) that adjusts waveform templates based on the recent accumulated mean shapes (1% of midline every 20 min). This allows for the same neuron to be tracked across consecutive days, as verified by the superimposition of waveforms acquired thoughout the experiment (Wavetracker software, Plexon Inc.).
(3 MB PPT).

Figure S6 Neuronal Yield Up to 159 neurons were recorded from three to four different brain areas.
(1.4 MB PPT).

Figure S7 Recording LFPs and Behaviors LFPs were recorded in parallel with spikes from the same electrodes. Neural signals were split, preamplified (1,000×), and filtered (0.5–400 Hz) by way of a Plexon LFP board. Signals were then fed to the MAP acquisition principal component through a NIDAQ card and digitized at 500 Hz. Behaviors were constantly recorded in videotape by two diametrically opposed infrared-sensitive CCD cameras (model WV-BP332, Panasonic, Laguna, Philippines). A millisecond-precision timer (model VTG-55, For-A Company, Tokyo, Japan) was used to synchronize the acquisition of spikes, LFPs, and videotape records.
Behavioral states were identified by the combined inspection of videotapes and the spectral content (1–20 Hz) of LFPs. Behaviors were classified according to the following criteria: (1) alert WK: active exploration with whisking, plus strong hippocampal θ rhythm; (2) quiet WK: stillness or grooming, with eyes open and low-power hippocampal θ rhythm; (3) SW sleep: stillness with eyes closed, plus large-amplitude hippocampal δ rhythm; (4) REM sleep: overall stillness with intermittent whisking, eyes closed, strong hippocampal θ rhythm.
The inspection of videotape records readily separates alert and quiet WK from sleep states, but the separation of SW and REM sleep relies strongly on LFP analysis. Hippocampal LFP is particularly useful to disambiguate SW and REM sleep: SW sleep has a strong δ band (2–4 Hz), while REM sleep shows increased θ band (5–8 Hz). The distinction between alert and quiet WK was used only for template selection (all templates taken from alert WK). For all other purposes, alert and quiet WK data were combined into a single WK category. The graphs depict the θ/δ hippocampal spectral ratios (mean ± SEM) of the three major behavioral states for rat 5 (entire recording).
(2.1 MB PPT).

Figure S8 Bin Size Exploration The figure shows the effect of using different bin sizes to calculate neuronal ensemble correlations. We observed quantitative differences (the larger the bin size, the larger the correlations), but qualitatively the correlations profiles are equivalent, i.e., have very similar shapes.
(404 KB PPT).

## Abbreviations

AP: anteroposterior
CSS: complex sensory stimulation
CX: cerebral cortex
DV: dorsoventral
HP: hippocampus
LFP: local field potential
ML: mediolateral
PU: putamen
REM: rapid eye-movement
SW: slow-wave
TH: thalamus
WK: waking
