# Supplementary material for: Long-Lasting Novelty-Induced Neuronal Reverberation during Slow-Wave Sleep in Multiple Forebrain Areas
Source: PLoS Biol. 2004 Jan 20;2(1):e24. doi: 10.1371/journal.pbio.0020024 (PMC314474; doi:10.1371/journal.pbio.0020024)
Supplement: Figure S1 — Four different objects were used to produce CSS. (9.7 MB PPT). [file pbio.0020024.sg001.ppt]

## Slide 1
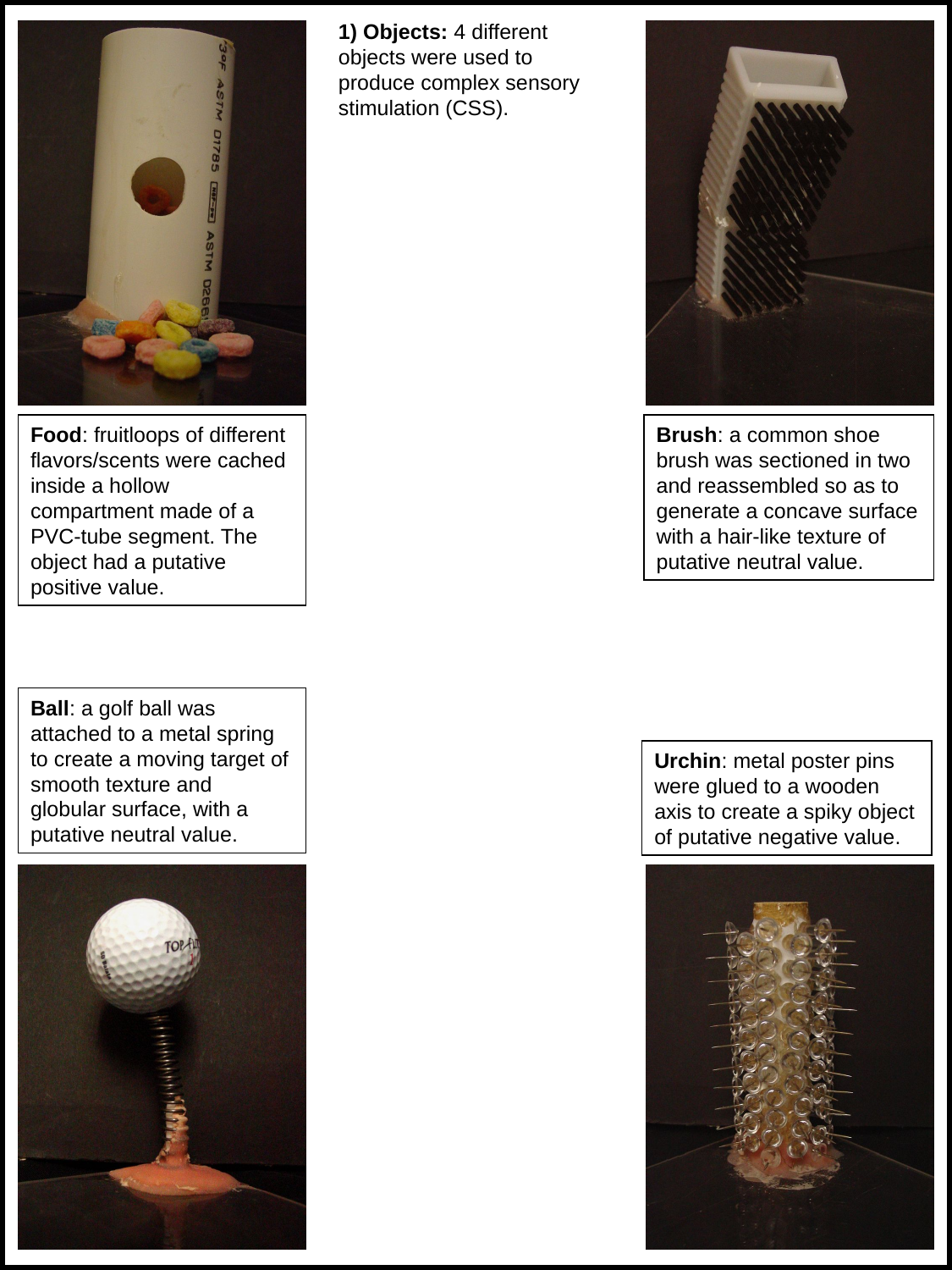

# 1) Objects: 4 different objects were used to produce complex sensory stimulation (CSS).
Food: fruitloops of different flavors/scents were cached inside a hollow compartment made of a PVC-tube segment. The object had a putative positive value.
Brush: a common shoe brush was sectioned in two and reassembled so as to generate a concave surface with a hair-like texture of putative neutral value.
Ball: a golf ball was attached to a metal spring to create a moving target of smooth texture and globular surface, with a putative neutral value.
Urchin: metal poster pins were glued to a wooden axis to create a spiky object of putative negative value.
