# Supplementary material for: Long-Lasting Novelty-Induced Neuronal Reverberation during Slow-Wave Sleep in Multiple Forebrain Areas
Source: PLoS Biol. 2004 Jan 20;2(1):e24. doi: 10.1371/journal.pbio.0020024 (PMC314474; doi:10.1371/journal.pbio.0020024)
Supplement: Figure S2 — All animals were highly habituated to the recording box, so that exposure to novel complex objects caused a general increase in the animals' arousal. Four of five animals showed an increase in time spent in WK with respect to SW and REM sleep during CSS (A), as compared to adjacent pre- and postnovelty periods of equal length (60 min). The only exception was rat 1, which showed nevertheless a marked exploratory drive, spending nearly 20% of the exposure period in direct whisker-contact with the objects (B). Individual object preferences were moderately varied, as indicated in (C). (746 KB PPT). [file pbio.0020024.sg002.ppt]

## Slide 1
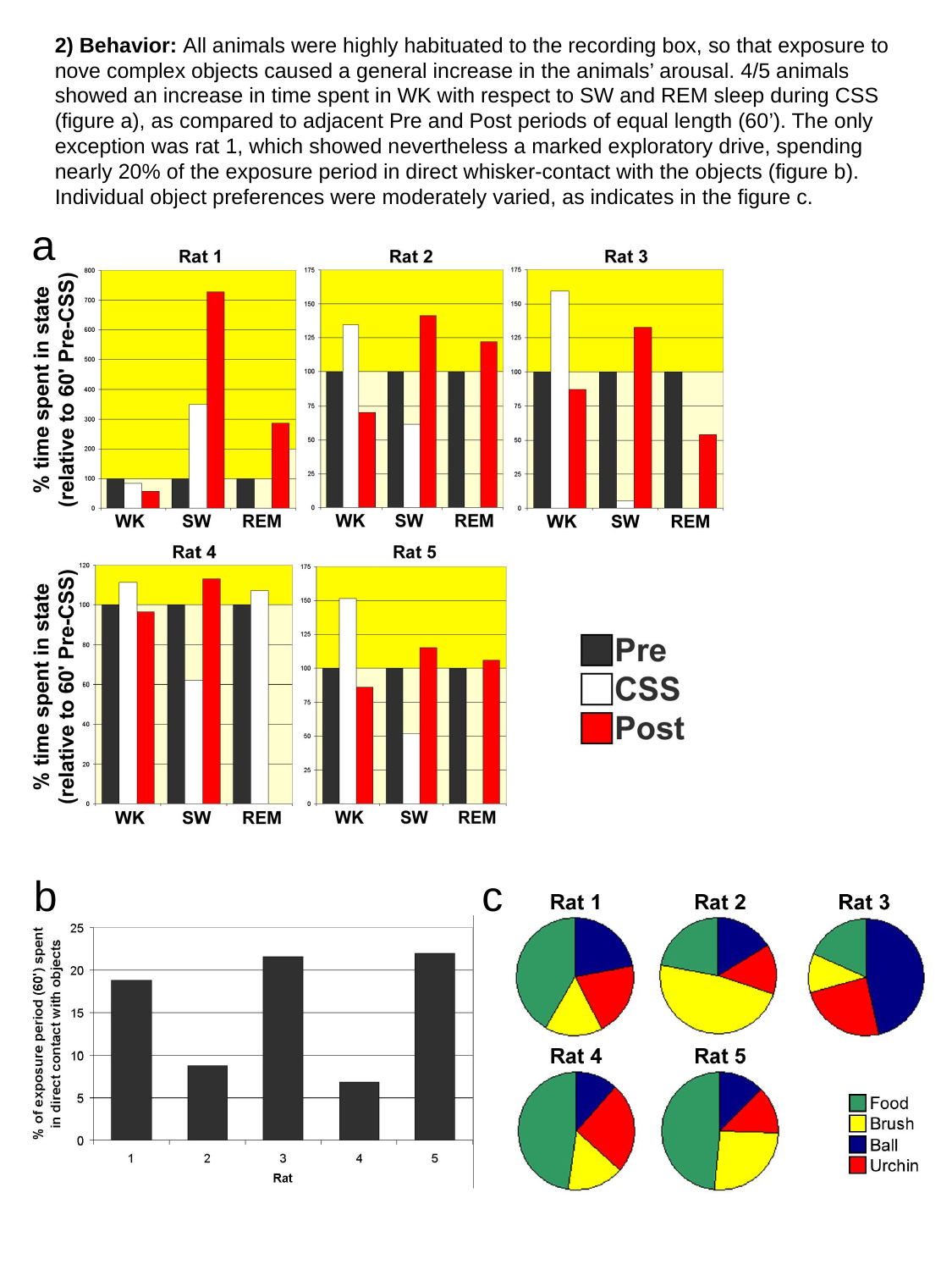

# 2) Behavior: All animals were highly habituated to the recording box, so that exposure to nove complex objects caused a general increase in the animals’ arousal. 4/5 animals showed an increase in time spent in WK with respect to SW and REM sleep during CSS (figure a), as compared to adjacent Pre and Post periods of equal length (60’). The only exception was rat 1, which showed nevertheless a marked exploratory drive, spending nearly 20% of the exposure period in direct whisker-contact with the objects (figure b). Individual object preferences were moderately varied, as indicates in the figure c.
a
b
c
