# Supplementary material for: Long-Lasting Novelty-Induced Neuronal Reverberation during Slow-Wave Sleep in Multiple Forebrain Areas
Source: PLoS Biol. 2004 Jan 20;2(1):e24. doi: 10.1371/journal.pbio.0020024 (PMC314474; doi:10.1371/journal.pbio.0020024)
Supplement: Figure S3 — Teflon-coated tungsten wires (50 μm diameter, 300 μm between wires, 1.0–1.2 MΩ at 1 KHz; California Fine Wire Company, Grover Beach, California, United States) were assembled in multielectrode arrays shaped to fit different neuroanatomical targets. (282 KB PPT). [file pbio.0020024.sg003.ppt]

## Slide 1
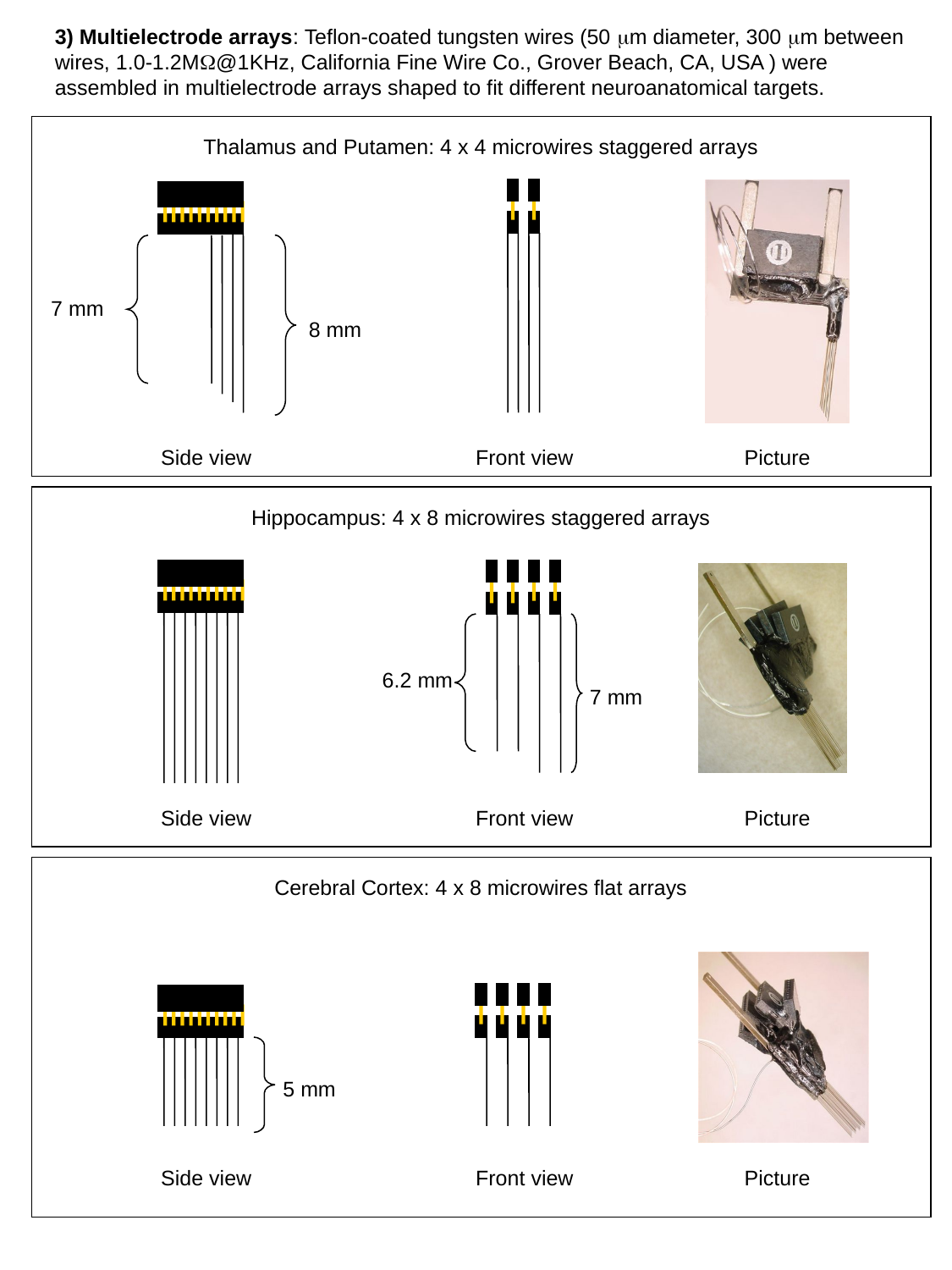

3) Multielectrode arrays: Teflon-coated tungsten wires (50 μm diameter, 300 μm between wires, 1.0-1.2MΩ@1KHz, California Fine Wire Co., Grover Beach, CA, USA ) were assembled in multielectrode arrays shaped to fit different neuroanatomical targets.
Thalamus and Putamen: 4 x 4 microwires staggered arrays
7 mm
8 mm
Side view Front view Picture
Hippocampus: 4 x 8 microwires staggered arrays
 6.2 mm
 7 mm
Side view Front view Picture
Cerebral Cortex: 4 x 8 microwires flat arrays
 5 mm
Side view Front view Picture
