# Supplementary material for: Long-Lasting Novelty-Induced Neuronal Reverberation during Slow-Wave Sleep in Multiple Forebrain Areas
Source: PLoS Biol. 2004 Jan 20;2(1):e24. doi: 10.1371/journal.pbio.0020024 (PMC314474; doi:10.1371/journal.pbio.0020024)
Supplement: Figure S4 — Frontal brain sections stained for cresyl-violet were used to determine the sites of electrode placement. Electrode tracks, tissue scars, and reference electrolytic lesions performed a few days before sacrifice were used to delimit the implant sites, indicated in red in the figure below. Numbers on the right represent standard AP coordinates (Paxinos and Watson 1997) in millimeters from Bregma. (6.3 MB PPT). [file pbio.0020024.sg004.ppt]

## Slide 1
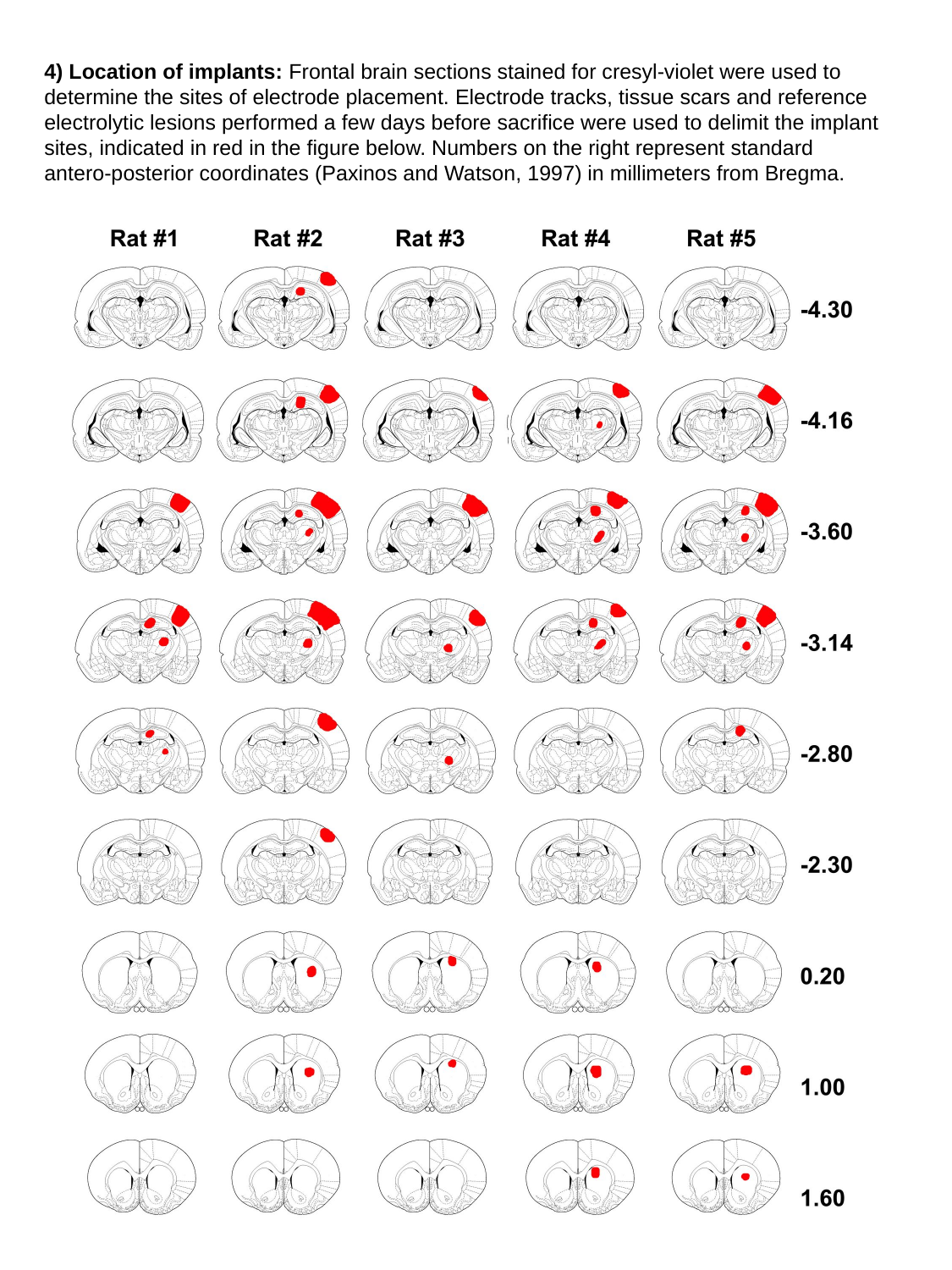

# 4) Location of implants: Frontal brain sections stained for cresyl-violet were used to determine the sites of electrode placement. Electrode tracks, tissue scars and reference electrolytic lesions performed a few days before sacrifice were used to delimit the implant sites, indicated in red in the figure below. Numbers on the right represent standard antero-posterior coordinates (Paxinos and Watson, 1997) in millimeters from Bregma.
