# Supplementary material for: Long-Lasting Novelty-Induced Neuronal Reverberation during Slow-Wave Sleep in Multiple Forebrain Areas
Source: PLoS Biol. 2004 Jan 20;2(1):e24. doi: 10.1371/journal.pbio.0020024 (PMC314474; doi:10.1371/journal.pbio.0020024)
Supplement: Figure S5 — To record neuronal activity, differentiated neural signal was preamplified (2,000×–32,000×) and digitized at 40 KHz. Up to four neuronal action potentials per recording channel were sorted online (SortClient 2002, Plexon Inc.) and validated by offline analysis (Offline Sorter 2.3, Plexon Inc.) according to the following cumulative criteria: voltage thresholds greater than two standard deviations of amplitude distributions; signal-to-noise ratio greater than 2.5 (as verified on the oscilloscope screen); less than 1% of interspike intervals smaller than 1.2 ms; and stereotypy of waveform shapes, as determined by a waveform template algorithm and principal component analysis. In order to continuously record individual neurons for up to 96 h, we used an adaptive algorithm (available on SortClient 2002, Plexon Inc.) that adjusts waveform templates based on the recent accumulated mean shapes (1% of midline every 20 min). This allows for the same neuron to be tracked across consecutive days, as verified by the superimposition of waveforms acquired thoughout the experiment (Wavetracker software, Plexon Inc.). (3 MB PPT). [file pbio.0020024.sg005.ppt]

## Slide 1
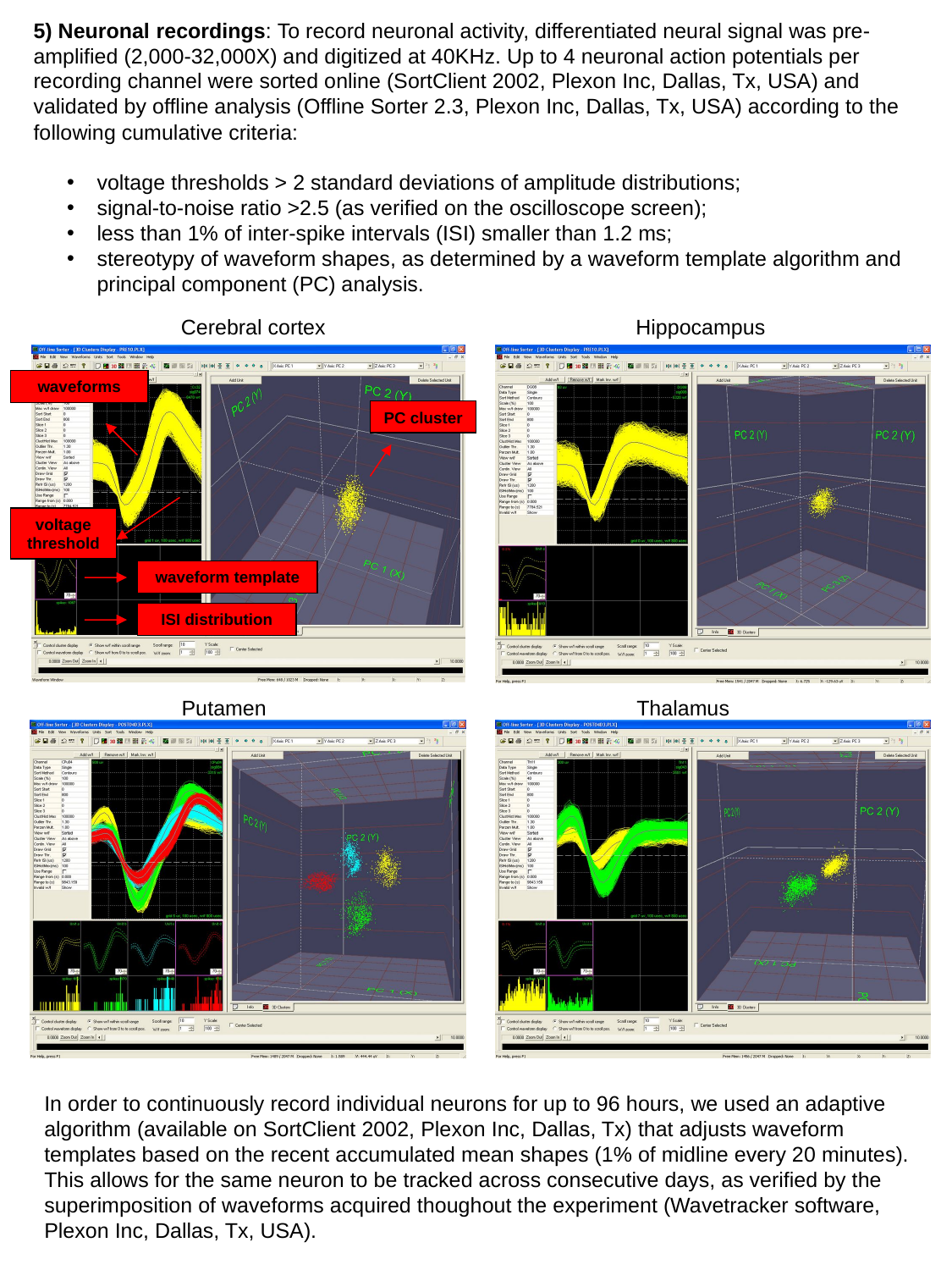

5) Neuronal recordings: To record neuronal activity, differentiated neural signal was pre-amplified (2,000-32,000X) and digitized at 40KHz. Up to 4 neuronal action potentials per recording channel were sorted online (SortClient 2002, Plexon Inc, Dallas, Tx, USA) and validated by offline analysis (Offline Sorter 2.3, Plexon Inc, Dallas, Tx, USA) according to the following cumulative criteria:
voltage thresholds > 2 standard deviations of amplitude distributions;
signal-to-noise ratio >2.5 (as verified on the oscilloscope screen);
less than 1% of inter-spike intervals (ISI) smaller than 1.2 ms;
stereotypy of waveform shapes, as determined by a waveform template algorithm and principal component (PC) analysis.
Cerebral cortex
Hippocampus
waveforms
PC cluster
voltage
threshold
waveform template
ISI distribution
Putamen
Thalamus
In order to continuously record individual neurons for up to 96 hours, we used an adaptive algorithm (available on SortClient 2002, Plexon Inc, Dallas, Tx) that adjusts waveform templates based on the recent accumulated mean shapes (1% of midline every 20 minutes). This allows for the same neuron to be tracked across consecutive days, as verified by the superimposition of waveforms acquired thoughout the experiment (Wavetracker software, Plexon Inc, Dallas, Tx, USA).
