# Supplementary material for: Long-Lasting Novelty-Induced Neuronal Reverberation during Slow-Wave Sleep in Multiple Forebrain Areas
Source: PLoS Biol. 2004 Jan 20;2(1):e24. doi: 10.1371/journal.pbio.0020024 (PMC314474; doi:10.1371/journal.pbio.0020024)
Supplement: Figure S6 — Up to 159 neurons were recorded from three to four different brain areas. (1.4 MB PPT). [file pbio.0020024.sg006.ppt]

## Slide 1
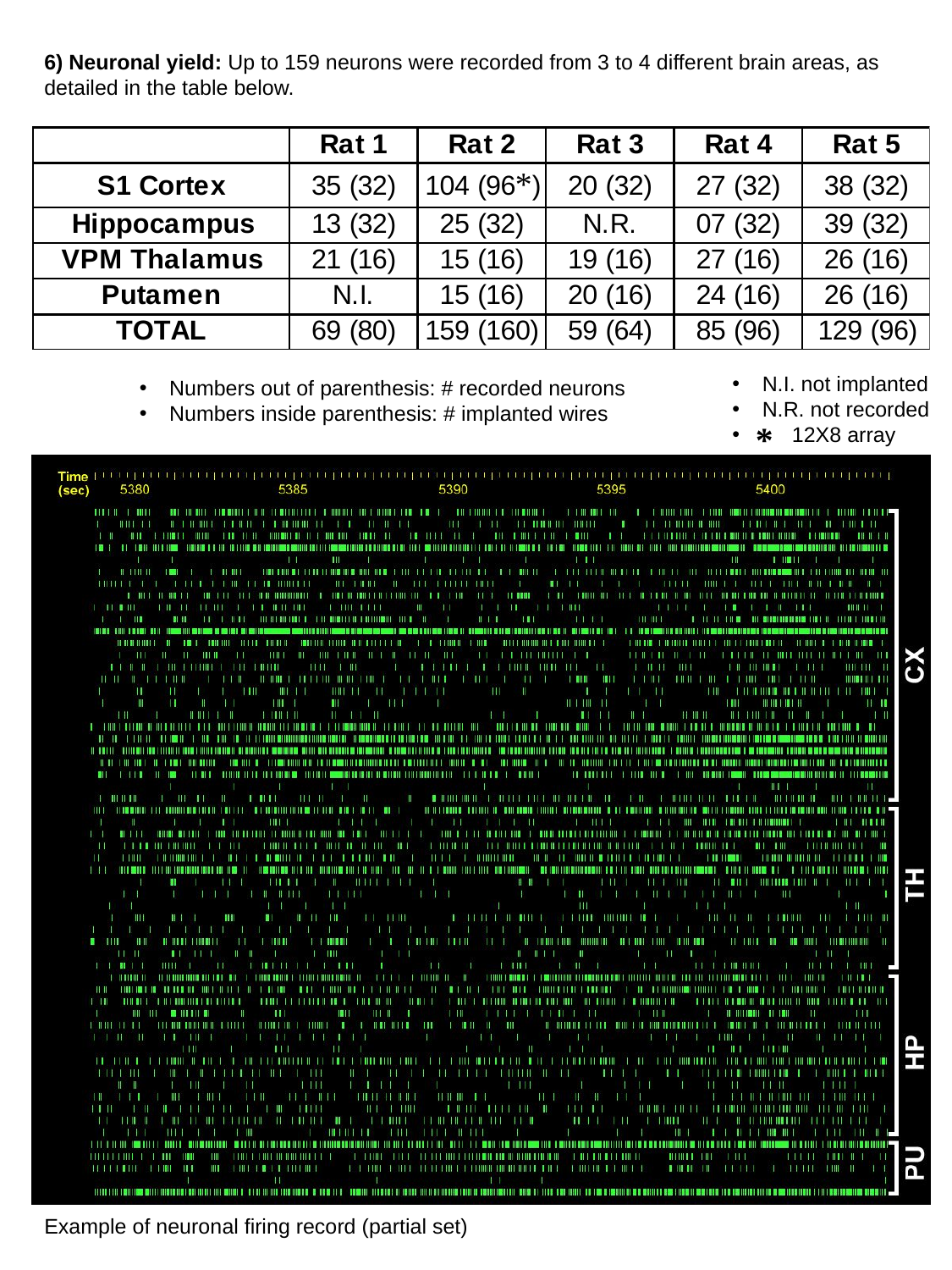

6) Neuronal yield: Up to 159 neurons were recorded from 3 to 4 different brain areas, as detailed in the table below.
N.I. not implanted
N.R. not recorded
 12X8 array
Numbers out of parenthesis: # recorded neurons
Numbers inside parenthesis: # implanted wires
*
Example of neuronal firing record (partial set)
