# Supplementary material for: Long-Lasting Novelty-Induced Neuronal Reverberation during Slow-Wave Sleep in Multiple Forebrain Areas
Source: PLoS Biol. 2004 Jan 20;2(1):e24. doi: 10.1371/journal.pbio.0020024 (PMC314474; doi:10.1371/journal.pbio.0020024)
Supplement: Figure S7 — LFPs were recorded in parallel with spikes from the same electrodes. Neural signals were split, preamplified (1,000×), and filtered (0.5–400 Hz) by way of a Plexon LFP board. Signals were then fed to the MAP acquisition principal component through a NIDAQ card and digitized at 500 Hz. Behaviors were constantly recorded in videotape by two diametrically opposed infrared-sensitive CCD cameras (model WV-BP332, Panasonic, Laguna, Philippines). A millisecond-precision timer (model VTG-55, For-A Company, Tokyo, Japan) was used to synchronize the acquisition of spikes, LFPs, and videotape records. Behavioral states were identified by the combined inspection of videotapes and the spectral content (1–20 Hz) of LFPs. Behaviors were classified according to the following criteria: (1) alert WK: active exploration with whisking, plus strong hippocampal θ rhythm; (2) quiet WK: stillness or grooming, with eyes open and low-power hippocampal θ rhythm; (3) SW sleep: stillness with eyes closed, plus large-amplitude hippocampal δ rhythm; (4) REM sleep: overall stillness with intermittent whisking, eyes closed, strong hippocampal θ rhythm. The inspection of videotape records readily separates alert and quiet WK from sleep states, but the separation of SW and REM sleep relies strongly on LFP analysis. Hippocampal LFP is particularly useful to disambiguate SW and REM sleep: SW sleep has a strong δ band (2–4 Hz), while REM sleep shows increased θ band (5–8 Hz). The distinction between alert and quiet WK was used only for template selection (all templates taken from alert WK). For all other purposes, alert and quiet WK data were combined into a single WK category. The graphs depict the θ/δ hippocampal spectral ratios (mean ± SEM) of the three major behavioral states for rat 5 (entire recording). (2.1 MB PPT). [file pbio.0020024.sg007.ppt]

## Slide 1
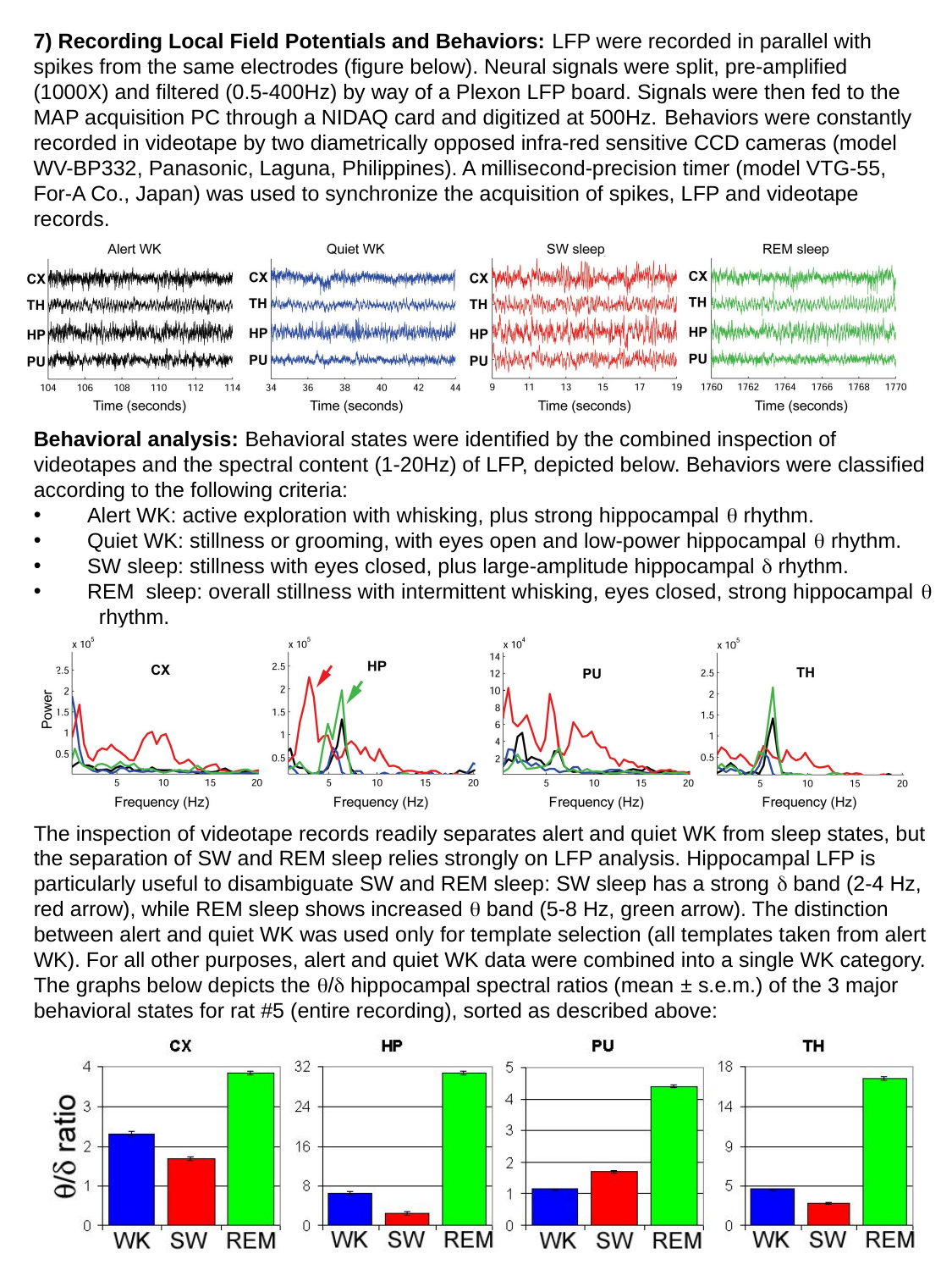

7) Recording Local Field Potentials and Behaviors: LFP were recorded in parallel with spikes from the same electrodes (figure below). Neural signals were split, pre-amplified (1000X) and filtered (0.5-400Hz) by way of a Plexon LFP board. Signals were then fed to the MAP acquisition PC through a NIDAQ card and digitized at 500Hz. Behaviors were constantly recorded in videotape by two diametrically opposed infra-red sensitive CCD cameras (model WV-BP332, Panasonic, Laguna, Philippines). A millisecond-precision timer (model VTG-55, For-A Co., Japan) was used to synchronize the acquisition of spikes, LFP and videotape records.
#
Behavioral analysis: Behavioral states were identified by the combined inspection of videotapes and the spectral content (1-20Hz) of LFP, depicted below. Behaviors were classified according to the following criteria:
 Alert WK: active exploration with whisking, plus strong hippocampal  rhythm.
 Quiet WK: stillness or grooming, with eyes open and low-power hippocampal  rhythm.
 SW sleep: stillness with eyes closed, plus large-amplitude hippocampal  rhythm.
 REM sleep: overall stillness with intermittent whisking, eyes closed, strong hippocampal  rhythm.
The inspection of videotape records readily separates alert and quiet WK from sleep states, but the separation of SW and REM sleep relies strongly on LFP analysis. Hippocampal LFP is particularly useful to disambiguate SW and REM sleep: SW sleep has a strong  band (2-4 Hz, red arrow), while REM sleep shows increased  band (5-8 Hz, green arrow). The distinction between alert and quiet WK was used only for template selection (all templates taken from alert WK). For all other purposes, alert and quiet WK data were combined into a single WK category. The graphs below depicts the / hippocampal spectral ratios (mean ± s.e.m.) of the 3 major behavioral states for rat #5 (entire recording), sorted as described above:
