# Supplementary material for: Long-Lasting Novelty-Induced Neuronal Reverberation during Slow-Wave Sleep in Multiple Forebrain Areas
Source: PLoS Biol. 2004 Jan 20;2(1):e24. doi: 10.1371/journal.pbio.0020024 (PMC314474; doi:10.1371/journal.pbio.0020024)
Supplement: Figure S8 — The figure shows the effect of using different bin sizes to calculate neuronal ensemble correlations. We observed quantitative differences (the larger the bin size, the larger the correlations), but qualitatively the correlations profiles are equivalent, i.e., have very similar shapes. (404 KB PPT). [file pbio.0020024.sg008.ppt]

## Slide 1
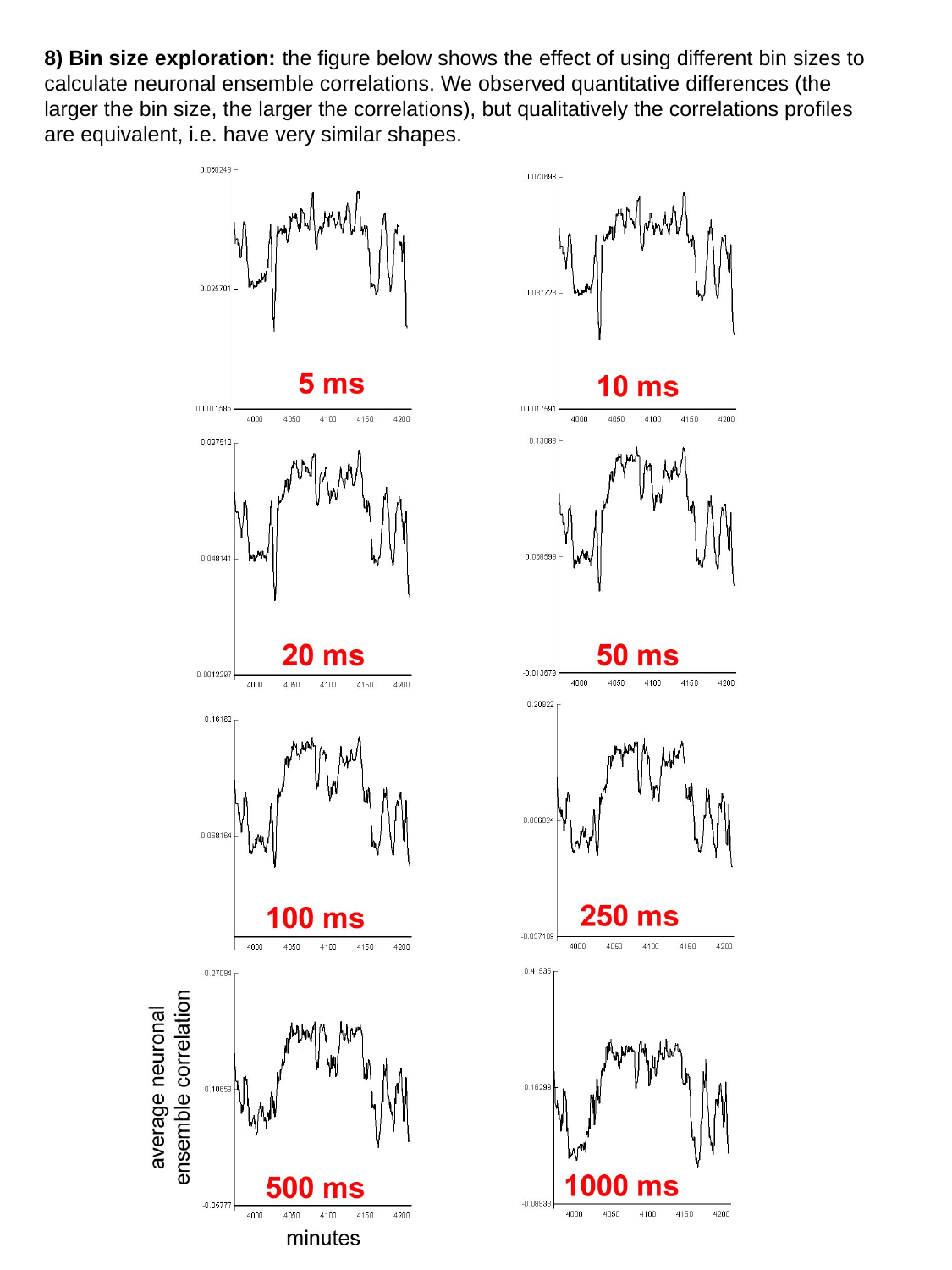

8) Bin size exploration: the figure below shows the effect of using different bin sizes to calculate neuronal ensemble correlations. We observed quantitative differences (the larger the bin size, the larger the correlations), but qualitatively the correlations profiles are equivalent, i.e. have very similar shapes.
